# Airborne Microbial Communities at High-Altitude and Suburban Sites in Toyama, Japan Suggest a New Perspective for Bioprospecting

**DOI:** 10.3389/fbioe.2019.00012

**Published:** 2019-02-05

**Authors:** Daisuke Tanaka, Kei Sato, Motoshi Goto, So Fujiyoshi, Fumito Maruyama, Shunsuke Takato, Takamune Shimada, Akihiro Sakatoku, Kazuma Aoki, Shogo Nakamura

**Affiliations:** ^1^Graduate School of Science and Engineering, University of Toyama, Toyama, Japan; ^2^Graduate School of Human and Environmental Studies, Kyoto University, Kyoto, Japan; ^3^JST/JICA, Science and Technology Research Partnership for Sustainable Development Program, Tokyo, Japan; ^4^Department of Microbiology, Graduate School of Medicine, Kyoto University, Kyoto, Japan

**Keywords:** atmosphere, bacteria, eukaryote, community, bioaerosol, bioprospecting

## Abstract

Airborne microorganisms, especially those at high altitude, are exposed to hostile conditions, including ultraviolet (UV) radiation, desiccation, and low temperatures. This study was conducted to compare the composition and abundance of airborne microorganisms at a high-altitude site, Mt. Jodo [2,839 m above mean sea level (AMSL)] and a suburban site (23 m AMSL) in Toyama, Japan. To our knowledge, this is the first study to investigate microbial communities in air samples collected simultaneously at two sites in relatively close proximity, from low and high altitude. Air samples were collected over a period of 3 years during 2009–2011. We then examined the bacterial and eukaryotic communities and estimated the abundance of bacteria and fungi with real-time TaqMan PCR. The airborne bacterial and eukaryotic communities differed between high-altitude and suburban sites on each sampling day. Backward trajectory analysis of air masses that arrived at high-altitude and suburban sites on each sampling day displayed almost the same paths. The bacterial communities were dominated by Actinobacteria, Firmicutes, and Proteobacteria, while the eukaryotic communities included Ascomycota, Basidiomycota, and Streptophyta. We also predicted some application of such microbial communities. The airborne bacterial and fungal abundance at the high-altitude site was about two times lower than that at the suburban site. These results showed that each airborne microbial communities have locality even if they are collected close location.

## Introduction

Airborne microbes are ubiquitous in the atmosphere, being present at a density of 10^3^–10^6^ cells per cubic meter of air. These microbes are emitted from terrestrial, soil, forest, desert, agricultural, and composting activities as well as urban, wetland, coastal, and marine environments (Jaenicke, 2005; Gandolfi et al., 2013). These organisms are exposed to hostile conditions, including scarcity of nutrients, UV radiation, desiccation, temperature and pH shifts, and the presence of reactive oxygen species. Physical and chemical characteristics of aerosols in the atmospheric boundary layer (ABL; from surface to about 1–2 km high) are distinct from those in the free troposphere just above the ABL. Airborne microbes play an important role in agriculture, the biosphere, cloud formation, global climate, and atmospheric dynamics (Jaenicke, 2005; Brodie et al., 2007; Després et al., 2007; Christner et al., 2008; Burrows et al., 2009). Moreover, these microbes provide a medium for the spread of diseases (Bowers et al., 2012; Cao et al., 2014; Fujiyoshi et al., 2017).

Studies of airborne bacteria at high altitudes conducted to date have mainly focused on bacterial ice nucleation activity (Matthias-Maser et al., 2000; Marinoni et al., 2004; Bowers et al., 2009; Vaïtilingom et al., 2012; Xu et al., 2017; Tandon et al., 2018). Bowers et al. (2012) presented the seasonal variability of airborne bacterial communities sampled from a pristine high-elevation atmospheric research site at Mt. Werner (3,220 m above sea level), CO, USA. DeLeon-Rodriguez et al. (2013) reported 17 bacterial taxa in the middle to upper troposphere across the United States, including taxa known to use C1–C4 carbon compounds present in the atmosphere. Recently, some studies have characterized airborne fungi occurring at high-altitude sites, focusing on those that are pathogens for humans and plants (Kumar and Attri, 2016; Pusz et al., 2017; Xu et al., 2017).

Here, we describe the bacterial and eukaryotic communities in air samples collected simultaneously from high-altitude and suburban sites in Toyama, central Japan, at five time points from September 2009 to September 2011 during late summer to early autumn. We previously reported seasonal variations in airborne bacterial community over a 1-year period at a suburban site in Toyama based on PCR-DGGE (Tanaka et al., 2015). In this study, we analyzed 16S and 18S rRNA gene hypervariable regions to characterize airborne microbial communities. Furthermore, quantitative PCR was used to estimate the total number of airborne bacteria and fungi in each sample. Finally, we discussed the potential for use of airborne microorganisms as a source for bioprospecting.

## Materials and Methods

### Air Samples

**Figure 1:**
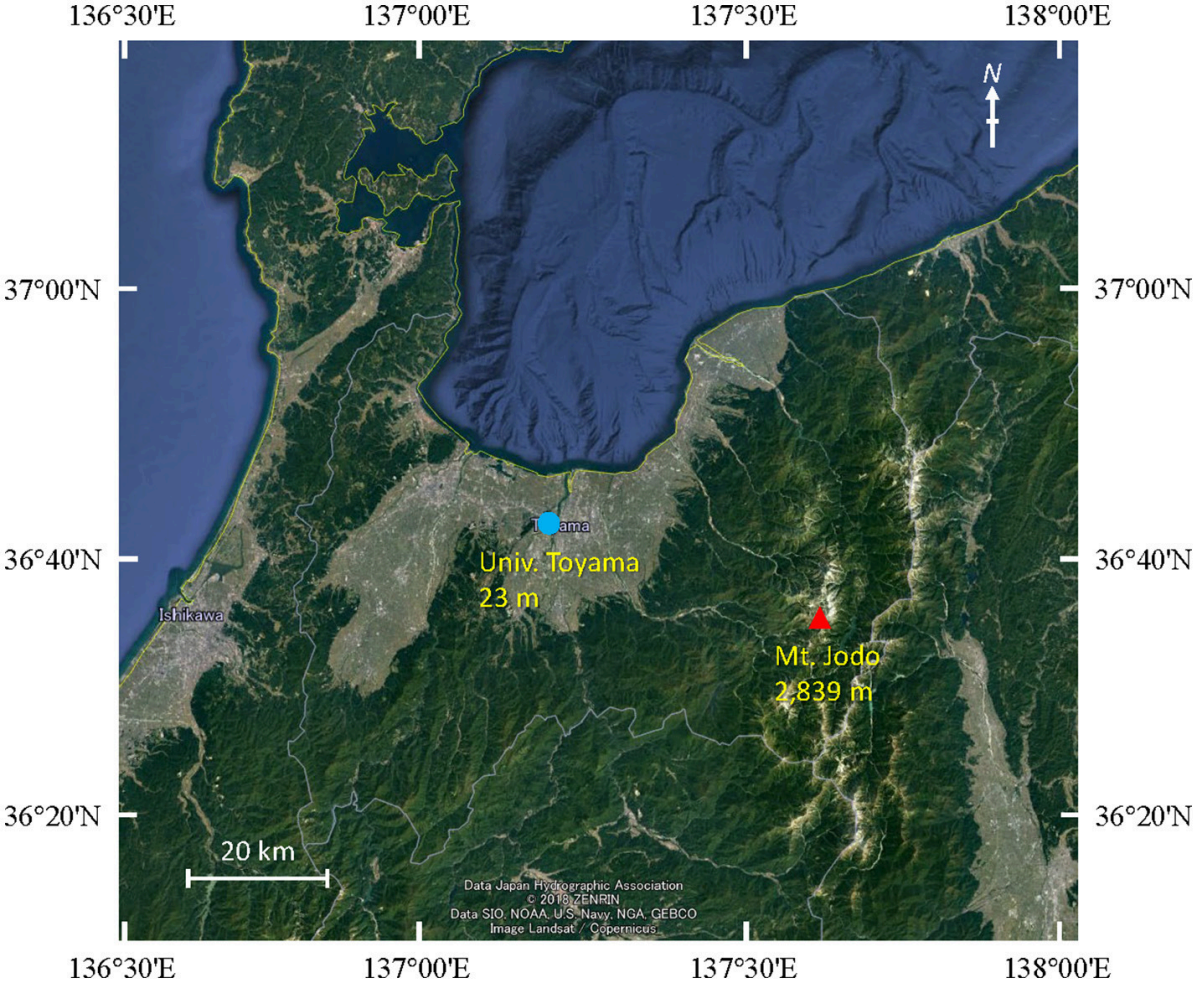
Map showing the sampling locations at Mt. Jodo (high-altitude) and Univ. Toyama (suburban) in Toyama, Japan.

Air samples were collected at five time points from 2009 to 2011 on August or September at two different sites in Toyama Prefecture, Japan. Toyama prefecture is surrounded by steep mountains on three sides and spreading fields. The size of the prefecture is 4,248 km^2^ (90 km east-west, 76 km north-south). The Toyama sampling sites include a suburban site on the roof of the three-story building of the Faculty of Science, University of Toyama (36°41′54″N, 137°11′13″E, 23 m above mean sea level, AMSL) and a high-altitude site at the peak of Mt. Jodo (36°34′00″N, 137°36′21″E, 2,839 m AMSL) (Figure 1). The two sites are located ~40 km apart from one another. Prior to the test, we evaluated the filtration efficiency using 0.2 or 0.4 μm pore size polycarbonate filters. Then, we decided to use 0.4 μm pore size filters. Similar pore size filter (pore size 0.45 μm) was also used in air sampling according to the procedure of Kobayashi et al. (2016). Air samples were collected by a vacuum pump using 47 mm diameter, 0.4 μm pore size polycarbonate filters (Advantec, Tokyo, Japan) at a flow rate of 5 L min^−1^ over 3 h (mostly 10:00–13:00). A total volume of 900 L of air was collected. All samples were collected when there was no precipitation. After sampling, the filters were stored at −20°C until DNA extraction. The duration of each sampling period and the corresponding weather conditions are presented in Table 1.

**Table 1:** Meteorological conditions during sampling.

**Date of collection**	**Location**	**Sample ID**	**Average temperature (^**°**^C)**	**Average relative humidity (%)**	**Average wind velocity (m/s)**	**Predominant wind direction**
9/16/2009	Mt. Jodo	20090916H	2.4	100	4.3	N
	Univ. Toyama	20090916S	22.9	64.5	2.2	NNE
8/20/2010	Mt. Jodo	20100820H	13.3	87.5	2.4	NNE
	Univ. Toyama	20100820S	33.3	65.1	2.1	NNE
9/24/2010	Mt. Jodo	20100924H	6.4	20.6	1.9	ESE
	Univ. Toyama	20100924S	21.1	63.6	6.1	NE
8/27/2011	Mt. Jodo	20110827H	11.4	95.3	2	W
	Univ. Toyama	20110827S	27.7	72.4	5.2	NNE
9/14/2011	Mt. Jodo	20110914H	13.2	85.5	1.3	SE
	Univ. Toyama	20110914S	30.7	59.5	3.1	NNE

### DNA Extraction and Illumina MiSeq Sequencing

The filtered samples were processed using an UltraClean Soil DNA isolation kit (MO BIO Laboratories, Carlsbad, CA, USA) according to the manufacturer's instructions. This kit uses a vigorous bead-beating method to effectively disrupt bacterial and fungal cells (Peccia and Hernandez, 2006). The purity and concentration of the extracted DNA was measured using a NanoDrop ND-1000 spectrophotometer (Nanodrop Technologies, Wilmington, DE, USA).

Subsequently, the V3–V4 region of the bacterial 16S rRNA gene was amplified using the 341F (5′-CCTACGGGNGGCWGCAG-3′) and 805R (5′-GACTACHVGGGTATCTAATCC-3′) primers (Klindworth et al., 2013). The V8 region of the eukaryotic 18S rRNA gene was amplified using the 1422–1440f (5′-ATAACAGGTCTGTGATGCC-3′) and 1624–1642r (5′-CGGGCGGTGTGTACAAAGG-3′) primers (Wang et al., 2014). For amplification of the 16S and 18S rRNA genes, samples were subjected to initial denaturation at 94°C for 2 min, followed by 35 cycles of denaturation at 94°C for 30 s, annealing at 55°C for 30 s, and extension at 72°C for 30 s, and then final extension at 72°C for 5 min (Klindworth et al., 2013). The amplified products were purified using Agencourt AMPure XP (Beckman Coulter, Brea, CA, USA) then, DNA quantification was conducted using Synergy H1 (Bio Tek, Tokyo, Japan) and a QuantiFluor dsDNA System (Promega, Madison, WI, USA). Purified amplicons were pooled in equimolar concentrations, then paired-end sequenced on an Illumina MiSeq instrument (Illumina, San Diego, CA, USA). The obtained sequence data were then processed using USEARCH version 10.0.240 and analyzed with the software package Quantitative Insights into Microbial Ecology (QIIME) version 1.9.1 (Caporaso et al., 2010). Sequences were clustered into operational taxonomic units (OTUs) using the Greengenes 13_8 reference OTU database (97% similarity). For 16S rRNA gene fragment analysis, chloroplast and mitochondrial OTUs were removed. Statistical analysis was conducted using the R software, version 3.2.0 (www.r-project.org). All sequences have been deposited in the DNA Data Bank of Japan (DDBJ) under the accession number DRA007352.

### Real-Time TaqMan PCR

Quantification of the bacterial 16S rRNA gene and fungal 18S rRNA gene was accomplished using real-time PCR. Bacterial DNA was quantified using the primers 1055f and 1392r and the TaqMan probe 16Staq1115 (Harms et al., 2003). Fungal DNA quantification was conducted using the primers FungiQuant-F and FungiQuant-R and the TaqMan probe FungiQuant-Prb (Liu et al., 2012). For fungi, universal fungal primers, and TaqMan probes covered the 1,199–1,549 *S. cerevisiae* numbering region of the 18S rRNA-encoding gene. Each reaction mixture was prepared in a total volume of 25 μL with 12.5 μL Premix Ex Taq (Probe qPCR, Takara Bio), 0.2 μM of each primer, 0.25 μM TaqMan probe, and 2 μL of standard or extracted DNA. For the assay, PCR amplification was performed in a Thermal Cycler Dice Real Time System (TP-850, Takara Bio, Otsu, Japan) under the conditions of initial denaturation for 30 s at 95°C followed by 40 cycles of 5 s at 95°C and 30 s at 60°C. DNA standards for bacteria and fungi were prepared from serial dilutions of the pGEM-T Easy Vector (Promega) containing the 16S rRNA gene from *Escherichia coli* and the 18S rRNA gene from *Cladosporium* sp., respectively. Duplicate aliquots of the standards and the samples were included in each PCR run and all assays included a negative control without template DNA.

### Backward Trajectory Analysis

To evaluate the process of air mass transport to the sample locations, backward trajectory analysis was conducted using the HYSPLIT model (https://www.ready.noaa.gov/HYSPLIT.php) developed by the National Oceanic and Atmospheric Administration (NOAA; Stein et al., 2015) with archived data from the Global Data Assimilation System (GDAS) meteorological dataset. Three-day backward trajectories were calculated for air masses arriving at the University of Toyama at heights of 500 m and 1,000 m and at the summit of Mt. Jodo at heights of 3,000 m and 3,500 m, respectively.

## Results and Discussion

### Meteorological Conditions and Backward Trajectories

As shown in Table 1, the temperature at the University of Toyama site was higher than that at Mt. Jodo. With the exception of September 24, 2010, the relative humidity at the high-altitude site was higher than that at the suburban site. The wind velocity at the high-altitude site and the suburban site was 1.3–4.3 and 2.1–6.1 m s^−1^, respectively. Additionally, the predominant wind direction was mainly north-northeast (NNE) and northeast (NE) at the suburban site, while no such tendency was observed at the high-altitude site. The predominant wind direction observed at the suburban site is concordant with our previous observations (Tanaka et al., 2015). There was no precipitation at each sampling time.

Three-day backward trajectories were calculated for air masses arriving at the high-altitude site at 3,000 and 3,500 m and at the suburban site at 500 and 1,000 m (Supplementary Figure 1). The two different trajectories for each site were similar on each sampling day except August 27, 2011. This similarity may have arisen because the two sites are not that far away from each other.

### Characterization of Airborne Bacterial Communities

During analysis of the bacterial community (bacterial 16S rRNA gene/V3–V4 region), we obtained 406,035 raw sequence reads from 10 samples (Table S1). The number of clean reads ranged from 2,404 to 20,794 (8,967/sample on average) in the high-altitude site samples and 19,888 to 33,701 (25,478/sample on average) in the suburban site samples, with a read length of 420 bp. There was a difference in the number of clean reads between high-altitude and suburban sites (*t*-test, *P* = 0.02). The number of OTUs ranged from 20 to 779 with an average of 266 OTUs per sample, and the number of OTUs in the suburban site samples was higher than in the high-altitude site samples (*t*-test, *P* = 0.03). The Chao1 index, Shannon index, and Simpson index were also higher in the suburban site samples (*t*-test, *P* = 0.003 for Chao1; *P* = 0.006 for Shannon; *P* = 0.04 for Simpson). Our results are possibly concordant with those reported by DeLeon-Rodriguez et al. (2013), who found that the upper troposphere harbors less complex communities than several other environments such as soils.

**Figure 2:**
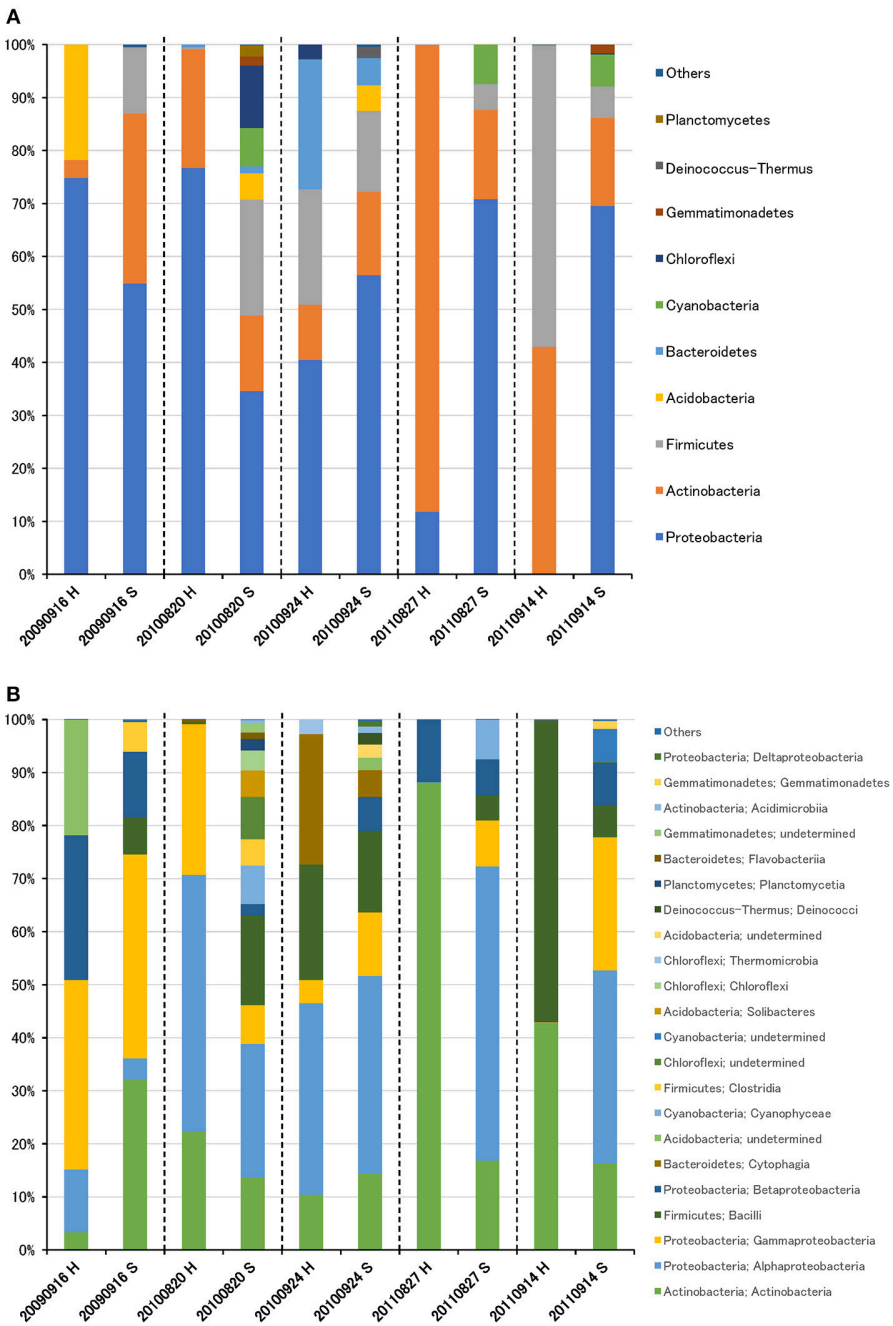
Taxonomic composition of bacterial reads at the phylum **(A)** and class **(B)** levels in air samples collected at high-altitude (H) and suburban (S) sites in Toyama.

The bacterial community was largely dominated by three phyla: Proteobacteria (49.1%), Actinobacteria (26.3%), and Firmicutes (14.0%) (Figure 2A). At the class level, the dominant groups were Actinobacteria (26.1%), Alphaproteobacteria (25.4%), Gammaproteobacteria (16.0%), Bacilli (12.9%), and Betaproteobacteria (7.5%) (Figure 2B). These findings are congruent with those reported in other air studies (Brodie et al., 2007; Fahlgren et al., 2010; Lee et al., 2010; Bowers et al., 2012; Maki et al., 2013; Tanaka et al., 2015; Xu et al., 2017), and hierarchical clustering of bacterial community did not show any clear trend (Supplementary Figures 2A, 3A).

### Characterization of Airborne Eukaryotic Communities

During analysis of the eukaryotic community (eukaryotic 18S rRNA gene/V8 region), we obtained 595,100 raw sequence reads from 10 samples (Table S2). The number of clean reads ranged from 36,467 to 53,771 (45,888/sample on average) in the high-altitude site samples and 40,124 to 70,933 (59,949/sample on average) in the suburban site samples, with a read length of 220 bp. There was a significant difference in the number of clean reads between high-altitude and suburban sites (*t*-test, *P* = 0.03). The number of OTUs ranged from 566 to 1,200 with an average of 825 OTUs per sample. Unlike the bacterial community, there were no significant differences in the number of OTUs, Chao1 index, Shannon index, or Simpson index of the eukaryotic community between the high-altitude and suburban site samples (*t*-test, *P* = 0.59 for OTUs; *P* = 0.68 for Chao1; *P* = 0.38 for Shannon; *P* = 0.08 for Simpson).

**Figure 3:**
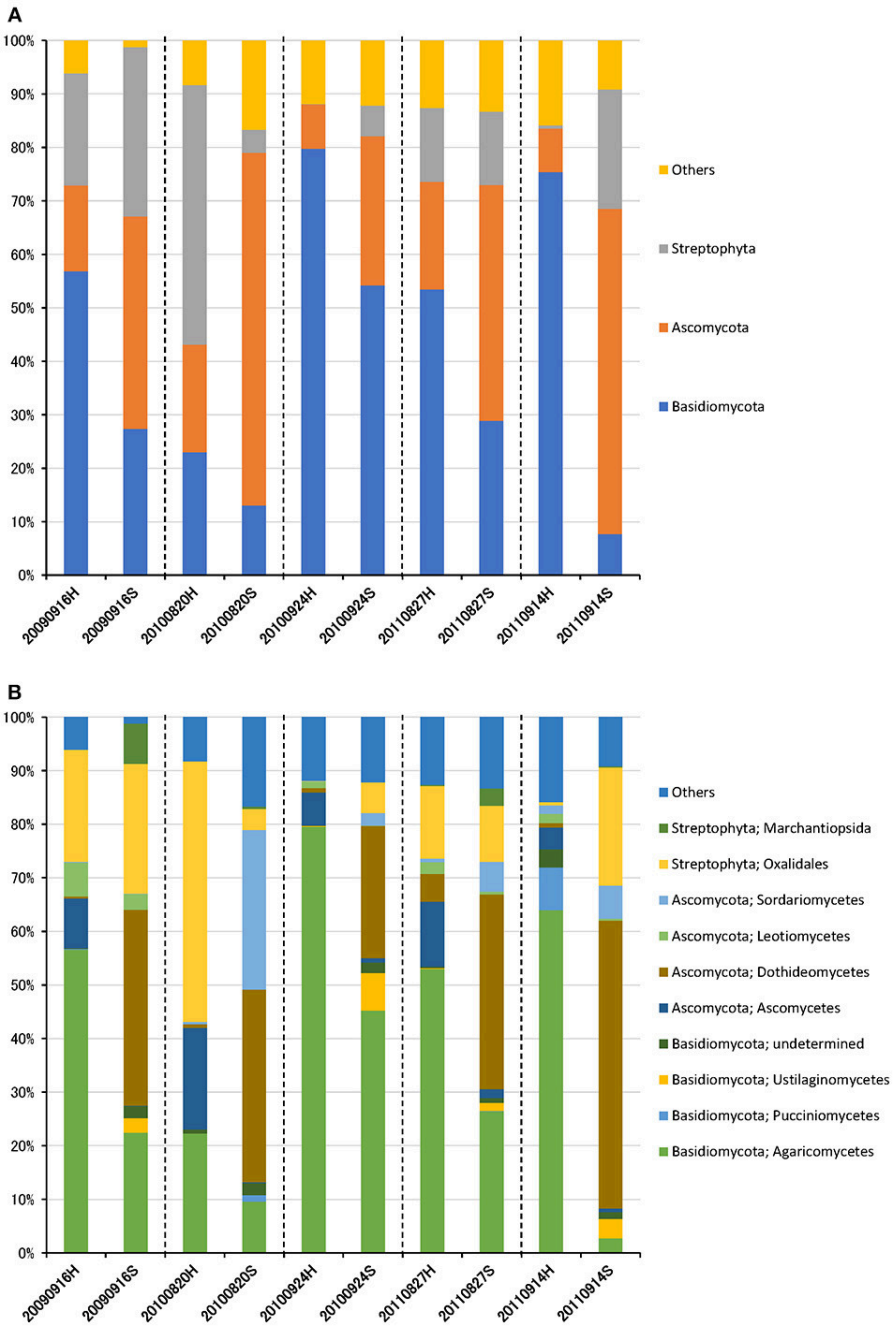
Taxonomic composition of eukaryotic reads at the phylum **(A)** and class **(B)** levels in air samples collected at high-altitude (H) and suburban (S) sites in Toyama.

**Figure 4:**
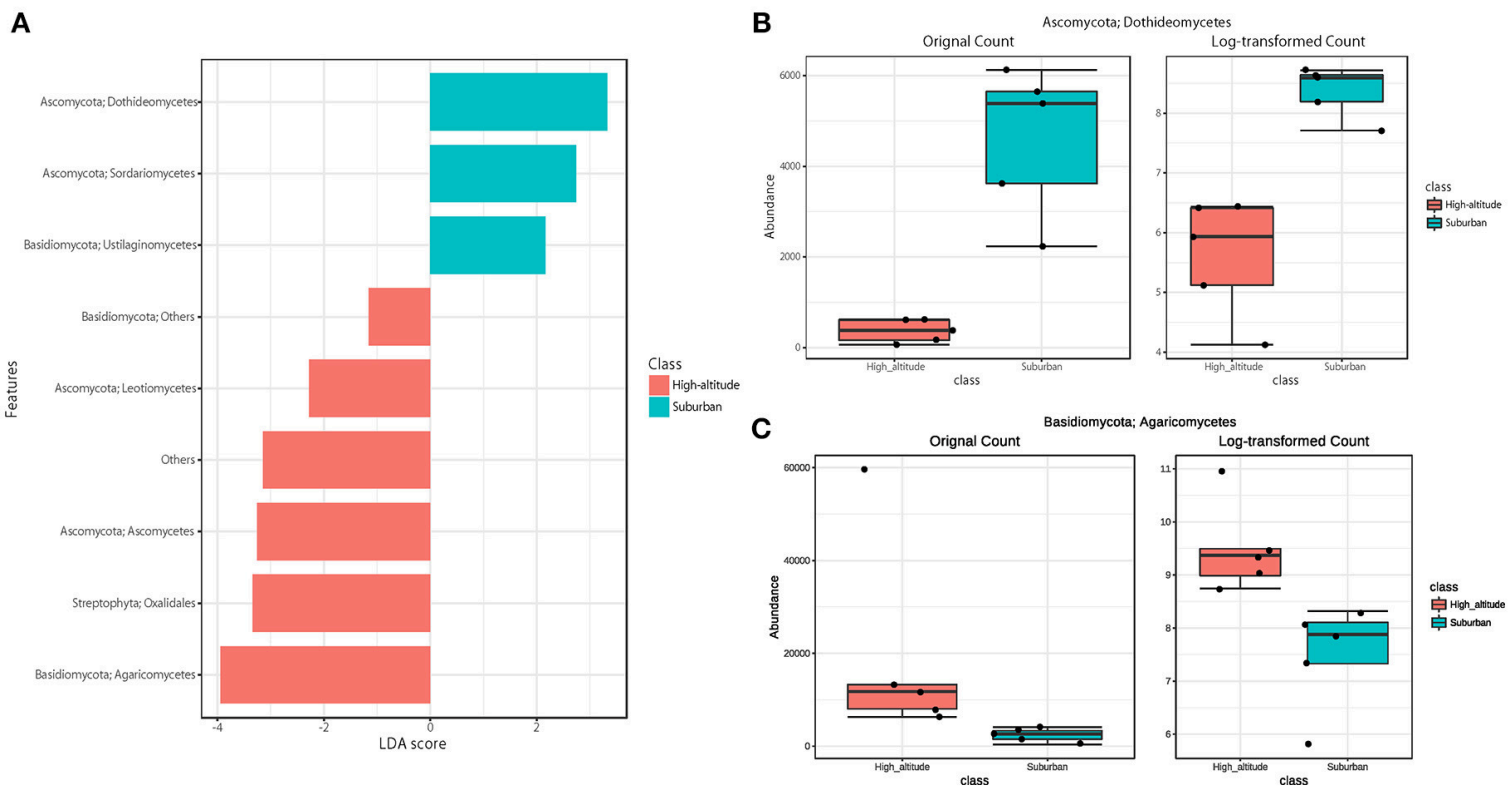
Linear discriminant analysis (LDA) effect size (LEfSe), a method for biomarker discovery, was used to determine eukaryotic taxa that best characterize each location. Differences in eukaryotic taxa between suburban and high-altitude (*P* < 0.05). **(A)** More abundant taxa in suburban are shown in blue, and in high-altitude are shown in red. The most different taxa in suburban **(B)** and in high-altitude **(C)** detected by the LEfSe analytic method.

The eukaryotic community was largely dominated by three phyla, Basidiomycota (41.7%), Ascomycota (30.9%), and Streptophyta (14.9%) (Figure 3A). This result is remarkably similar to previous work using a multidomain PCR primer set that was designed to capture a portion of the small-subunit rRNA gene from Archaea, Bacteria, and Eukarya (Bowers et al., 2013). At the class level, the dominant groups were Agaricomycetes (37.9%), Dothideomycetes (19.4%), Magnoliopsida (14.9%), Ascomycetes (5.3%), and Sordariomycetes (4.6%) (Figure 3B). It is widely accepted that Dothideomycetes is the most represented taxa in Ascomycetes, while Agaricomycetes is within the Basidiomycetes group (Yamamoto et al., 2012; Dannemiller et al., 2014; Shin et al., 2015; Núñez et al., 2016; Woo et al., 2018). Dothideomycetes, which includes genera associated with allergenic fungi such as *Alternaria, Epicoccum, Curvularia*, and *Cladosporium*, comprised almost half of the Ascomycota (Shin et al., 2015). Agaricomycetes includes many types of mushroom species, and generally do not contain described allergenic or pathogenic members, with the exception of some Agaricales (Yamamoto et al., 2012; Haga et al., 2014). In this study, differences in the eukaryotic community composition between high-altitude and suburban sites were observed (Figure 3B, Supplementary Figures 2B, 3B). Agaricomycetes (Basidiomycota) was richer in the high-altitude samples than the suburban samples, while Dothideomycetes (Ascomycota) was more abundant at the suburban site samples (Figure 4).

### Quantification of Airborne Microorganisms

**Figure 5:**
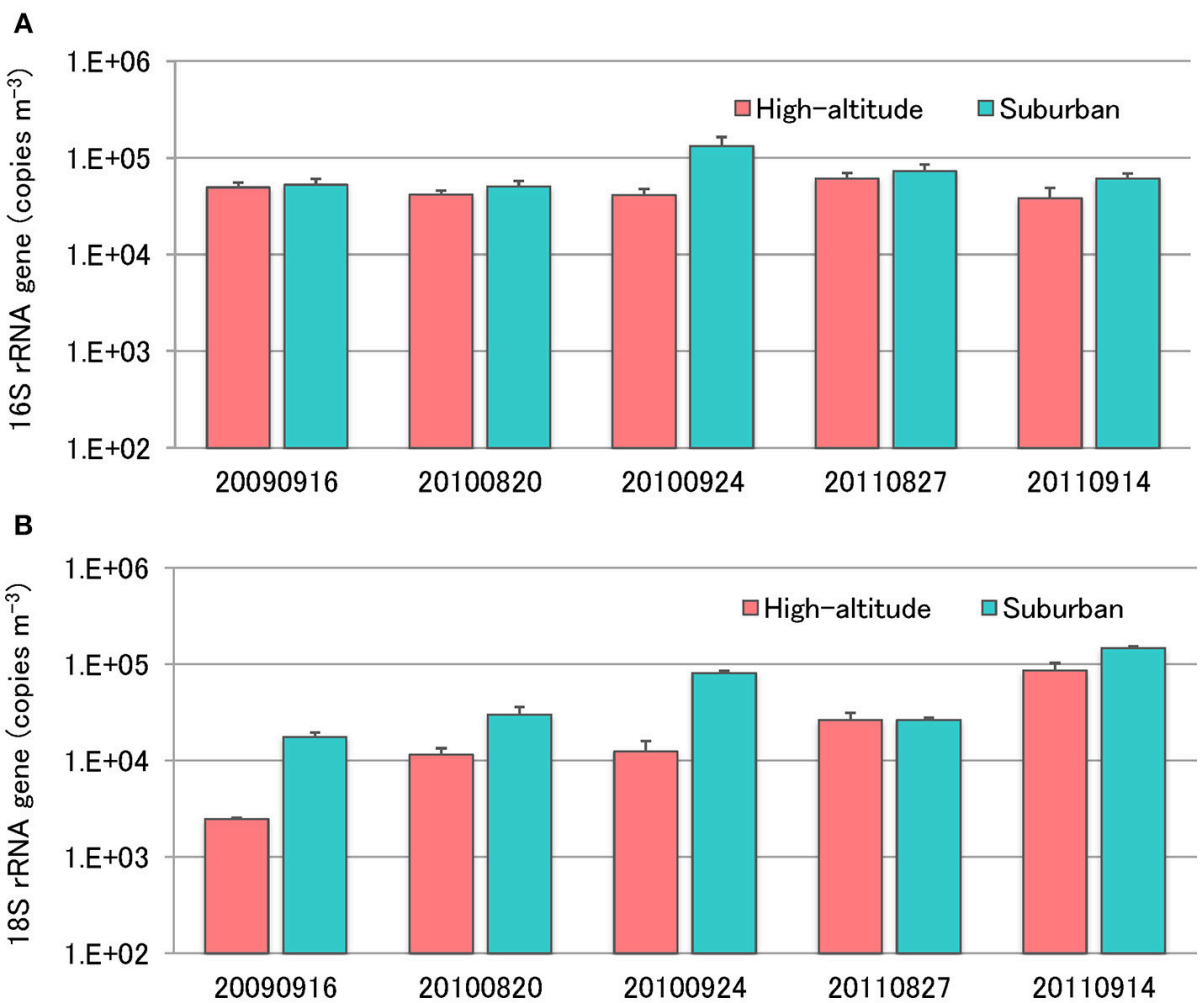
Quantification of total bacterial 16S rRNA genes **(A)** and fungal 18S rRNA genes **(B)** using real-time TaqMan PCR.

Enumeration of total bacteria and total fungi was conducted using quantitative real-time TaqMan PCR analysis of the bacterial 16S rRNA gene and the fungal 18S rRNA gene (Figure 5). The number of bacteria in air samples collected from Mt. Jodo and the University of Toyama ranged from 3.8 × 10^4^ to 6.1 × 10^4^ copies m^−3^ (4.6 × 10^4^ copies m^−3^ on average) and from 5.1 × 10^4^ to 1.3 × 10^5^ copies m^−3^ (7.4 × 10^4^ copies m^−3^ on average), respectively (Figure 5A). The number of fungi in air samples from the high-altitude site and the suburban site ranged from 2.5 × 10^3^ to 8.6 × 10^4^ copies m^−3^ (2.8 × 10^4^ copies m^−3^ on average) and from 1.8 × 10^4^ to 1.5 × 10^5^ copies m^−3^ (6.0 × 10^4^ copies m^−3^ on average), respectively (Figure 5B). This study showed that airborne bacterial and fungal abundance at the high-altitude site was about two times lower than that at the suburban site. The total bacteria and total fungi observed in this study are similar to those reported in other airborne microbial studies (Lee et al., 2010; Li et al., 2010; Bertolini et al., 2013; DeLeon-Rodriguez et al., 2013; Dannemiller et al., 2014; Gandolfi et al., 2015). Assuming an average rRNA gene copy number of four per bacterial genome and 30–100 per fungal genome (DeLeon-Rodriguez et al., 2013), our results suggest that bacterial abundance is one or two orders of magnitude greater than that of fungi at the high-altitude and suburban sites. These results are concordant with previous quantitative measurements using molecular and microscopic approaches (Bauer et al., 2002; DeLeon-Rodriguez et al., 2013; Gabey et al., 2013).

### Differences of High-Altitude and Suburban Microbial Community

**Figure 6:**
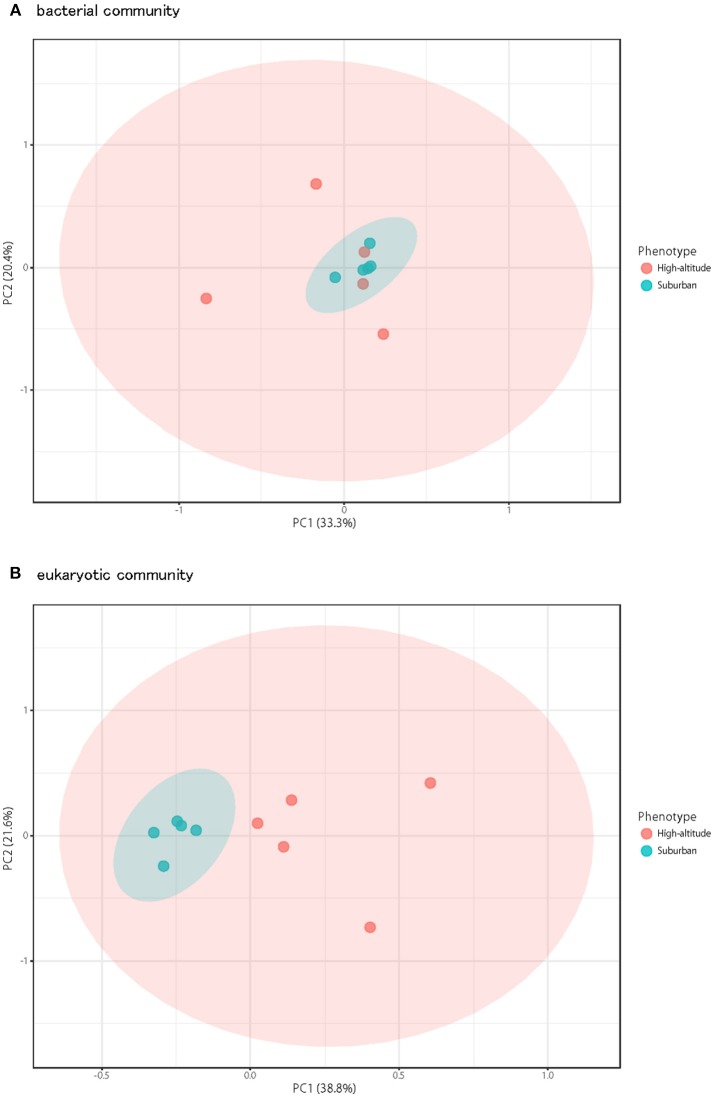
Principal coordinates plot shows overall variation in bacterial community **(A)** and eukaryotic community **(B)**. Five samples from the high-altitude (red) and suburban (blue) groups were plotted with two coordinates. The mean and standard deviation in each axis are indicated by an ellipse for each located group.

Although there was no significant difference in bacterial communities between the high-altitude and suburban sites, different taxons were detected in the eukaryotic community such as Agaricomycetes (Basidiomycota) at the high-altitude site and Dothideomycetes (Ascomycota) at the suburban site (Figures 2–4, Supplementary Figures 2B, 3B). Among abundant 18S OTUs, the dominant genera in Dothideomycetes included *Cladosporium* sp. (6.3–41.2% in the suburban samples and 0.1–2.4% in the high-altitude samples; 11.6% on average) and *Dothideomycetes* sp. (2.4–19.9% in the suburban samples and 0.1–1.5% in the high-altitude samples; 5.4% on average) (Table S4). Although *Cladosporium* is generally considered the most common outdoor genus in temperate climates (Gonçalves et al., 2010; Núñez et al., 2016; Lin et al., 2018), this fungus was only detected in low percentages from the high-altitude sites. In addition, the two different trajectories for each site were similar on each sampling day except August 27, 2011. The main cause may be related to vegetation type and weather conditions (Awad, 2005; Lin et al., 2018). Therefore, these findings suggest that the influence of local environmental factors on the airborne eukaryotic community are more important than those on the airborne bacterial community. Relationships between bacterial communities in samples from the two locations were shown by principal coordinates analysis (Figure 6A). Bacterial communities at the suburban site were more clustered than at the high-altitude site. These results were also observed in eukaryotic communities (Figure 6B). These findings suggest that the bacterial and eukaryotic communities at the high-altitude site fluctuate more than at the suburban site.

### Potential Applications of Airborne Microorganisms

The top 30 abundant 16S rRNA OTUs found during analysis of the airborne bacterial community made up more than 70.9% of all sequences (Table S3). Among these OTUs, bacteria in the order Actinomycetales (3 OTUs) and *Bacillus* sp. (1 OTUs) can be considered a useful source for antimicrobial compounds (Motta et al., 2007; Weber and Werth, 2015). The cosmopolitan distribution of Actinomycetales and *Bacillus* sp. may be partly a result of their ability to form spores, which can travel long distances in the air (Hervàs et al., 2009). *Methylobacterium* sp. (3 OTUs) can be used to reduce environmental contamination because they are able to degrade toxic compounds, tolerate high heavy metal concentrations, and increase plant tolerance to these compounds (Dourado et al., 2015). Moreover, these bacteria have genes related to plant-bacteria interactions that may be important for developing strains able to promote plant growth and protection against phytopathogens, showing its agricultural importance. Additionally, *Pseudomonas* sp. (3 OTUs), *Streptomyces* sp. (1 OTUs), *Bradyrhizobium* sp. (1 OTU), and *Hymenobacter* sp. (1 OTU) can be a source of natural pigments (Narsing Rao et al., 2017). On the other hand, the 30 most abundant 18S OTUs found upon analysis of the airborne eukaryotic community made up more than 88.2% of all sequences (Table S4). *Aspergillus* sp. (1 OUT) can be a source of natural pigments (Narsing Rao et al., 2017). Smets et al. (2016) found that airborne bacteria were useful for biotechnological applications because of their unique metabolic enzymes and metabolites as well as their ability to resist typical airborne conditions such as drought, UV irradiation, specific pollutants, and low temperatures. As described above, detected airborne microorganisms showed differences between high-altitude and suburban sites. These situations should be considered at the time of screening of beneficial airborne microorganisms.

## Author Contributions

DT conceived and designed the experiments. DT, KS, and ST performed the experiments. DT, MG, SF, and FM analyzed and interpreted the data. DT, SF, and FM wrote the paper. DT, SF, FM, MG, TS, AS, KA, and SN reviewed drafts of the manuscript.

### Conflict of Interest Statement

The authors declare that the research was conducted in the absence of any commercial or financial relationships that could be construed as a potential conflict of interest.
