# Supplementary material for: Airborne Microbial Communities at High-Altitude and Suburban Sites in Toyama, Japan Suggest a New Perspective for Bioprospecting
Source: Front Bioeng Biotechnol. 2019 Feb 5;7:12. doi: 10.3389/fbioe.2019.00012 (PMC6370616; doi:10.3389/fbioe.2019.00012)
Supplement: Supplementary file 5 [file Data_Sheet_1.docx]

Supplementary Material

Airborne microbial communities at high-altitude and suburban sites in Toyama, Japan suggest a new perspective for bioprospecting

Daisuke Tanaka^1^*, Kei Sato^1^, Motoshi Goto^1^, So Fujiyoshi^2, 3^, Fumito Maruyama^3, 4^, Shunsuke Takato^1^, Takamune Shimada^1^, Akihiro Sakatoku^1^, Kazuma Aoki^1^, Shogo Nakamura^1^

^1^Graduate School of Science and Engineering, University of Toyama, Toyama, Japan,

^2^Graduate School of Human and Environmental Studies, Kyoto University, Kyoto, Japan,

^3^JST/JICA, Science and Technology Research Partnership for Sustainable Development Program, Tokyo, Japan

^4^Department of Microbiology, Graduate School of Medicine, Kyoto University, Kyoto, Japan,

*Corresponding author: tanakada@sci.u-toyama.ac.jp


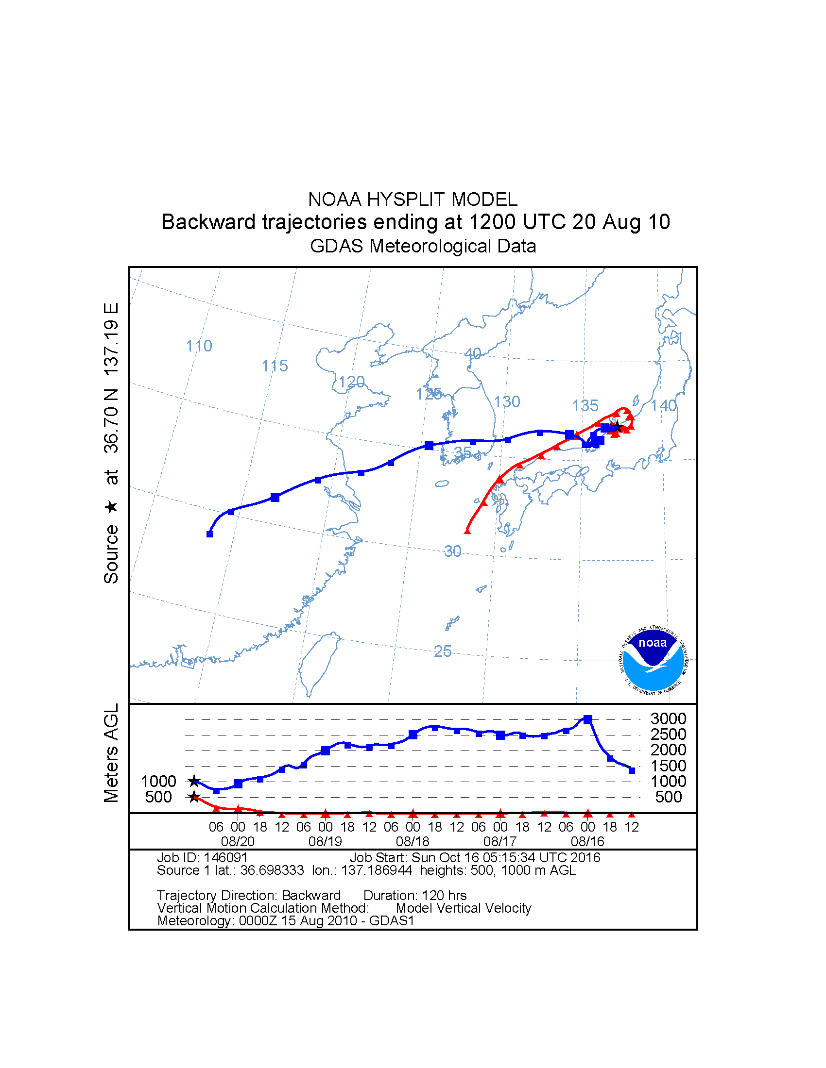

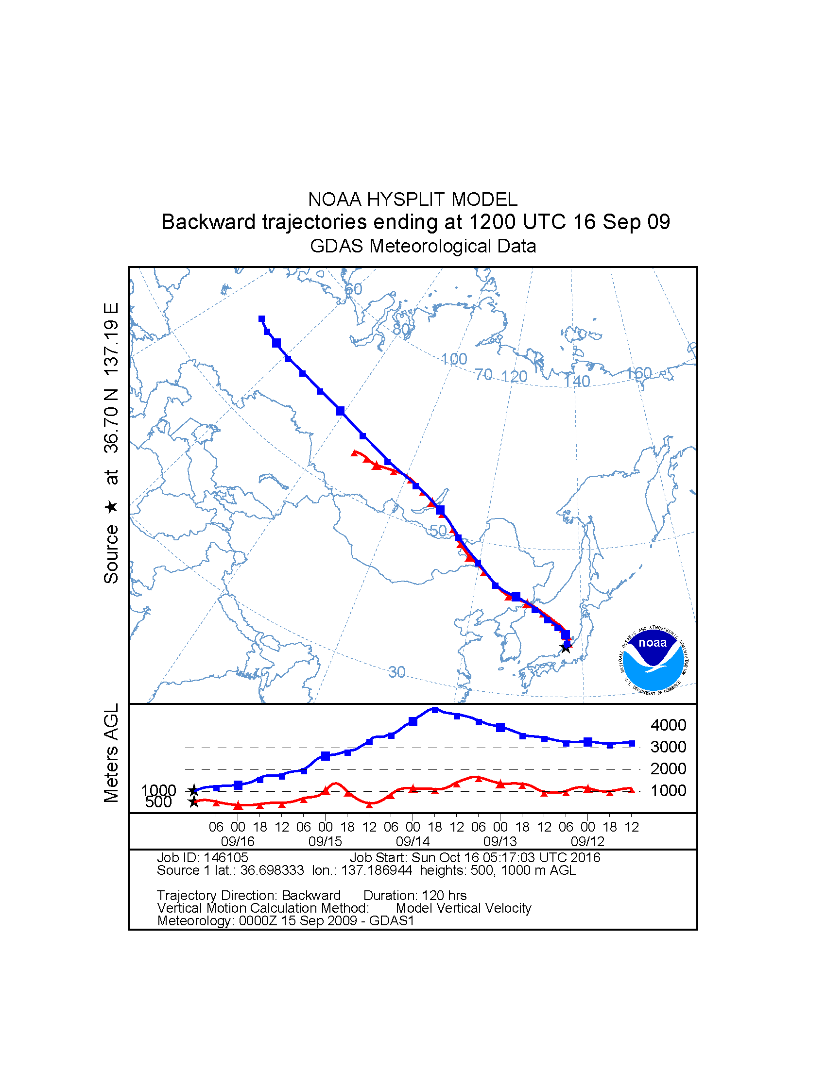

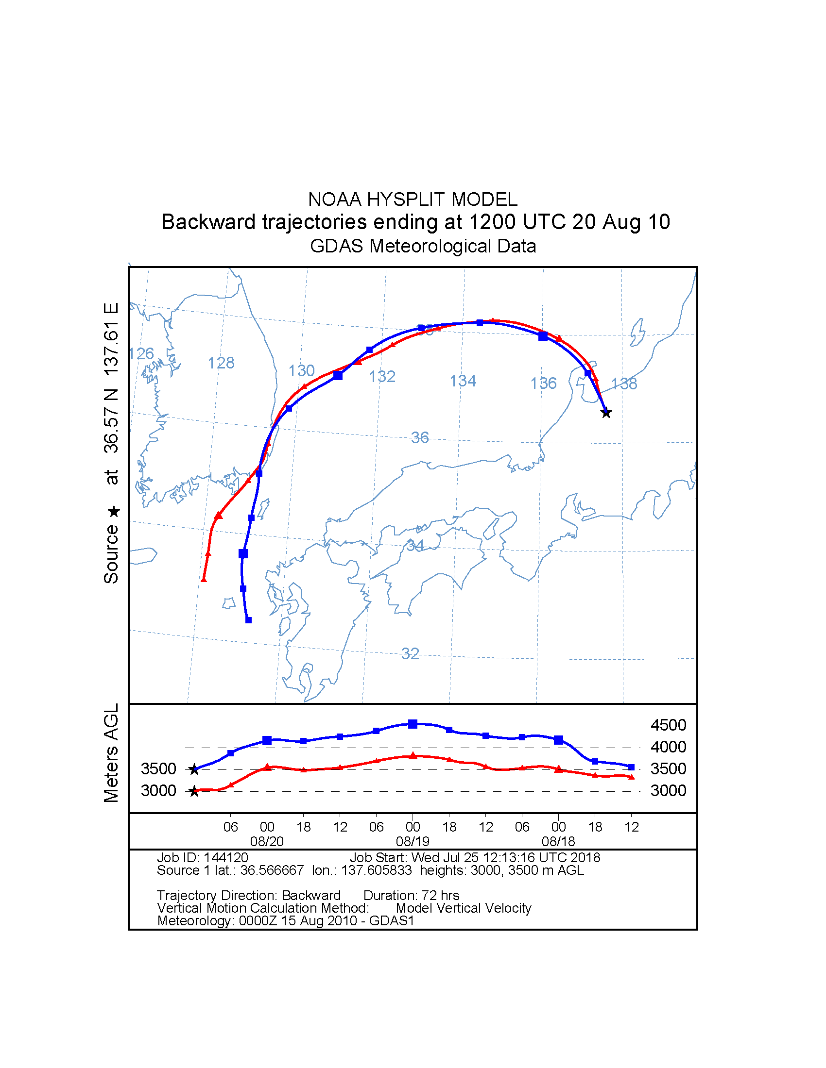

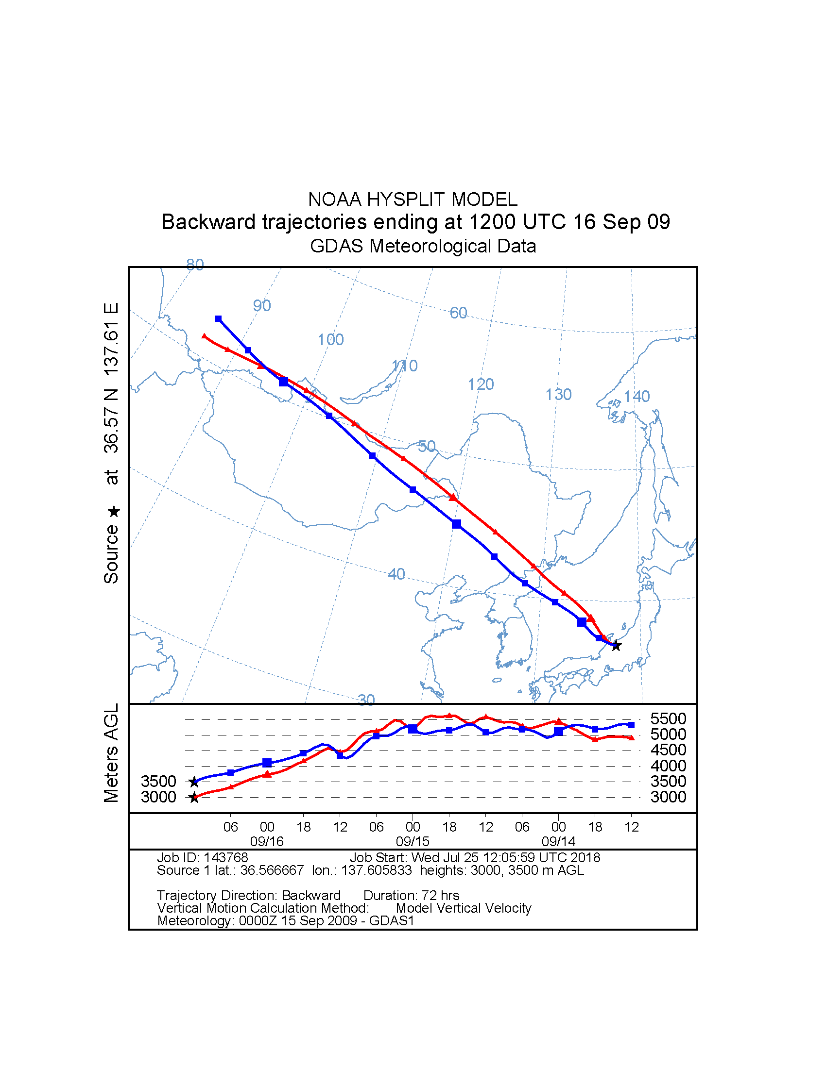


(A)

(B)

(C)

(D)

**Supplementary Figure 1.** Hierarchial clustering based on the Bray-Curtis dissimilarity index.

**Supplementary Figure 1.** Backward trajectories calculated with the NOAA HYSPLIT model (Stein et al., 2015) for air masses arriving at the summit of Mt. Jodo (A, C, E, G, I) at heights of 3000 m (red) and 3500 m (blue) and at the University of Toyama (B, D, F, H, J) at heights of 500 m (red) and 1000 m (blue).


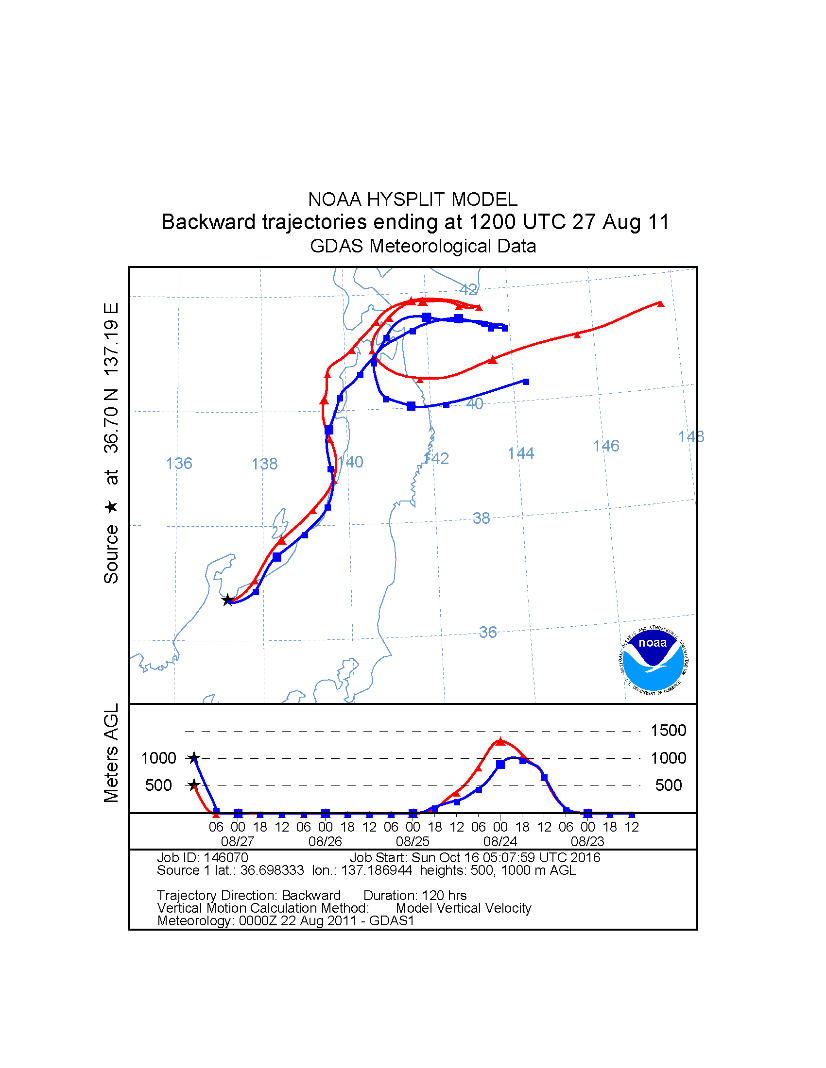

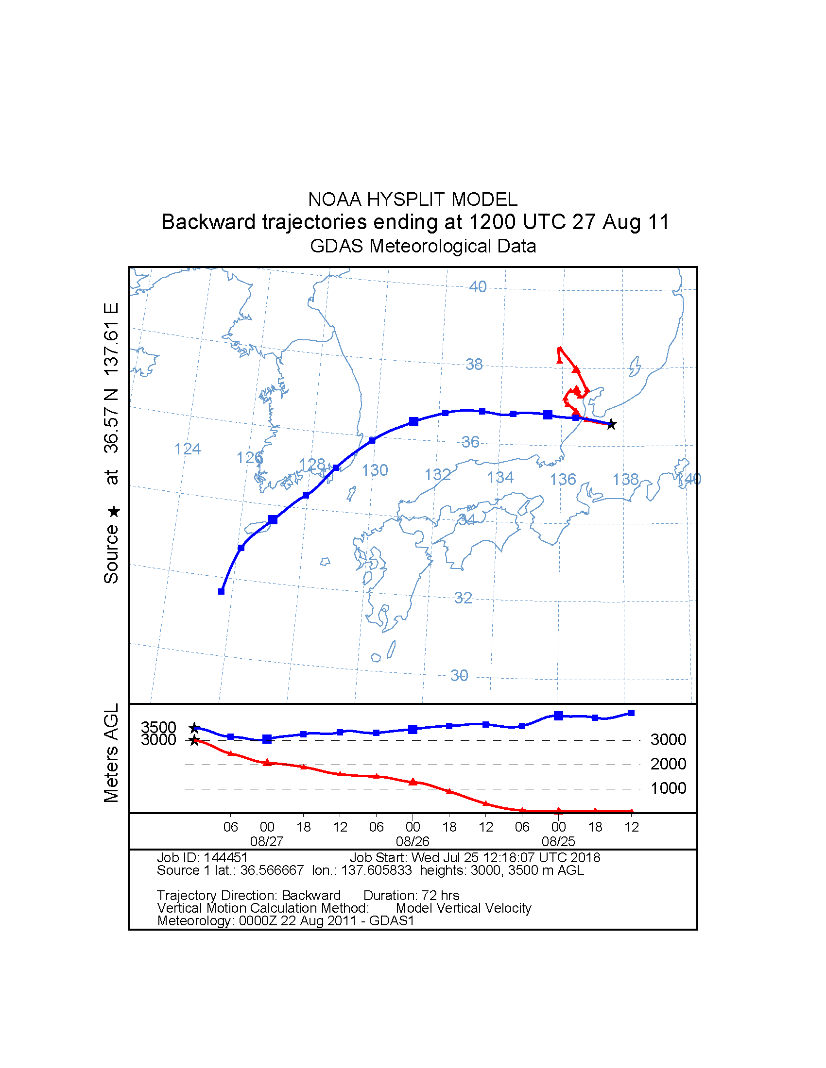

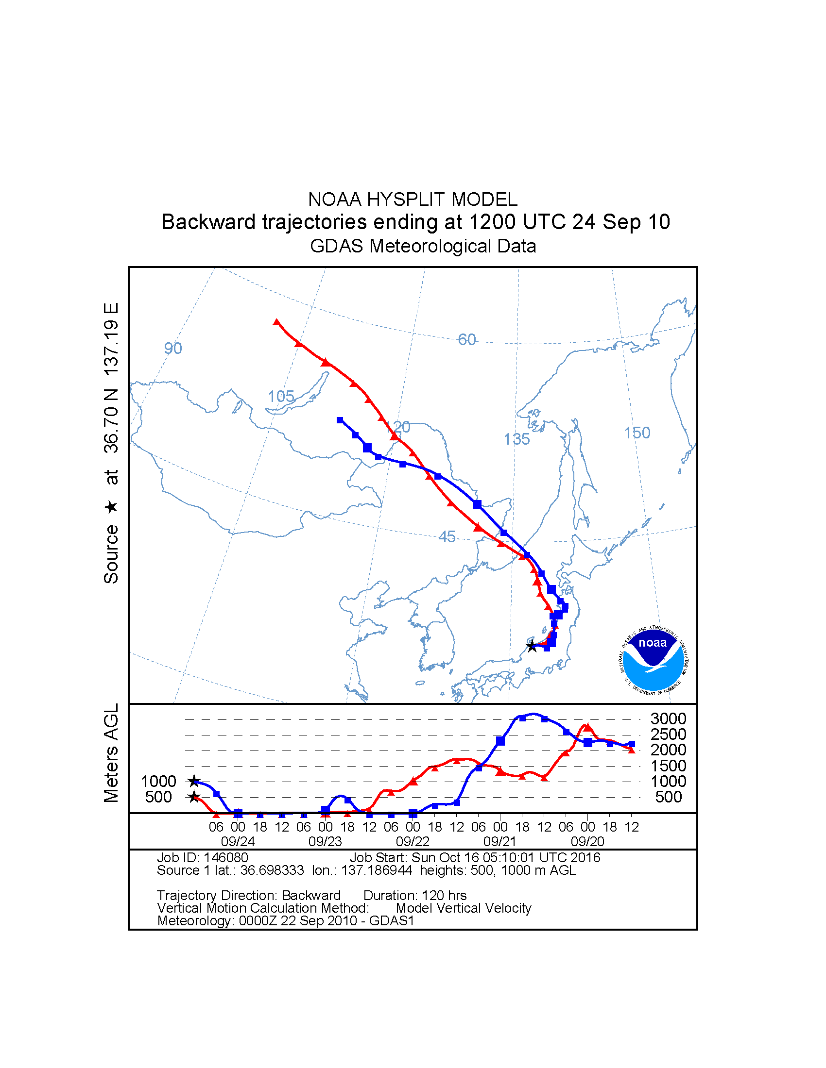

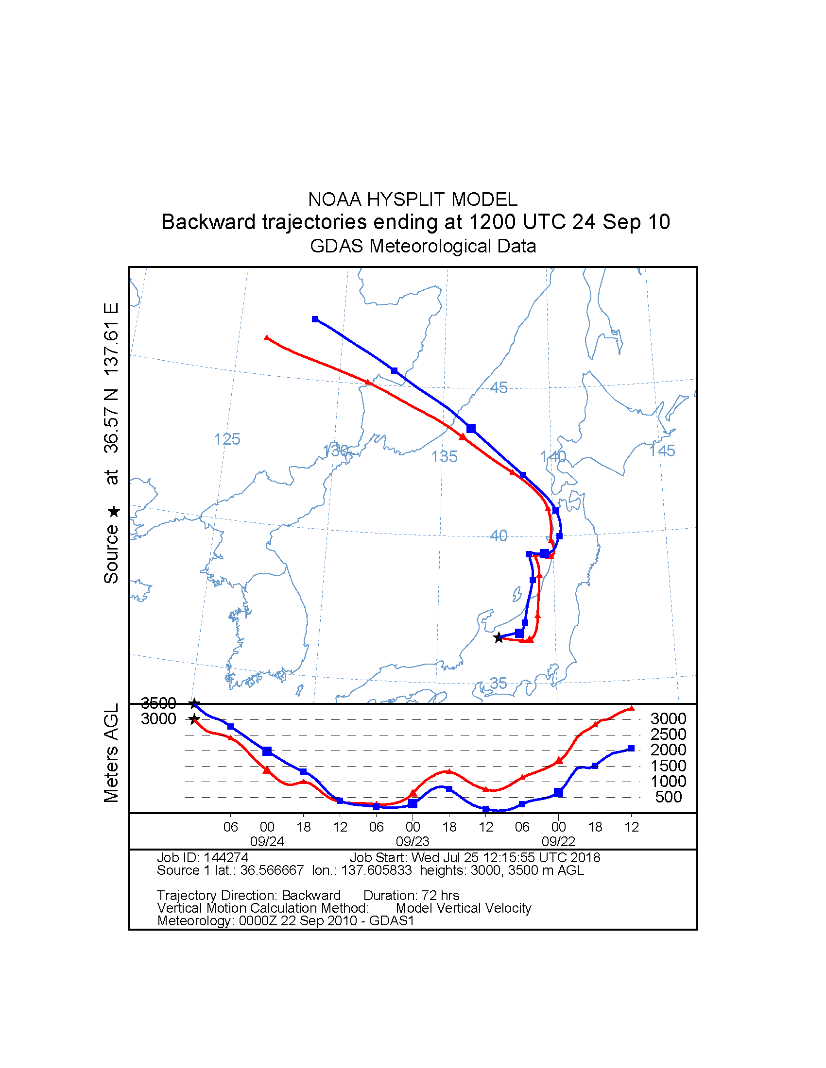


(H)

(G)

(F)

(E)

Fi.1

**Supplementary Figure 1. (continued)**


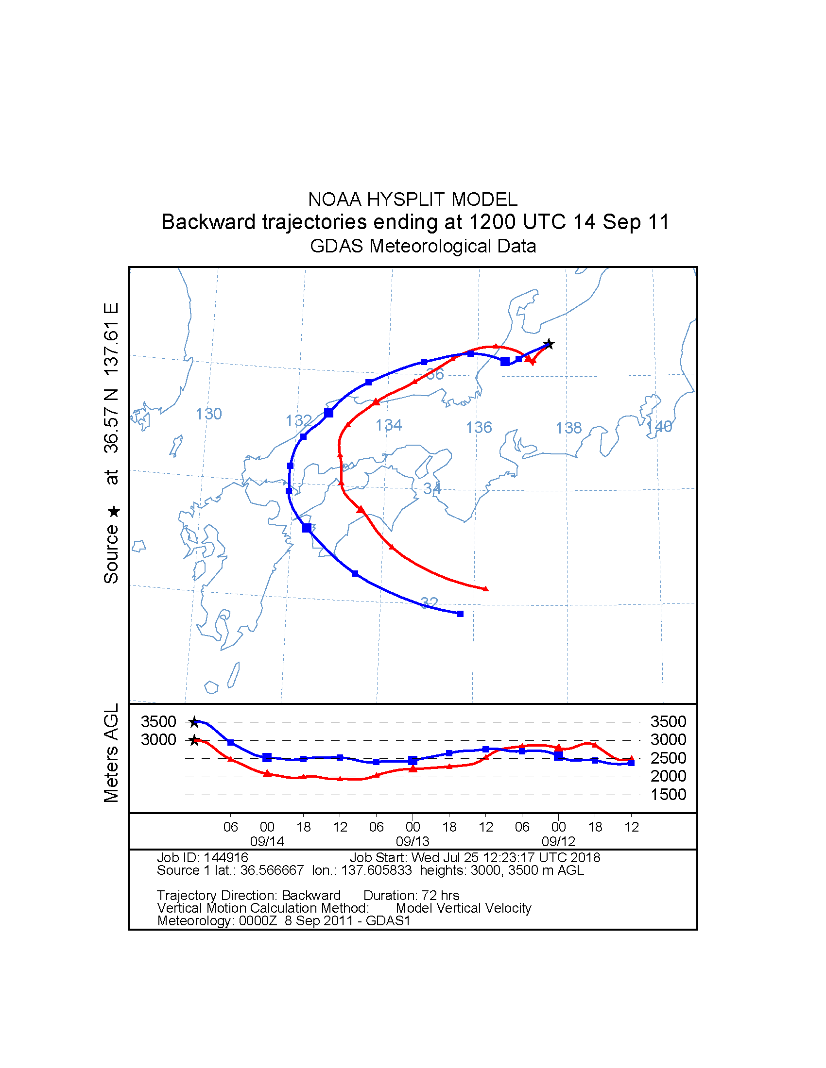

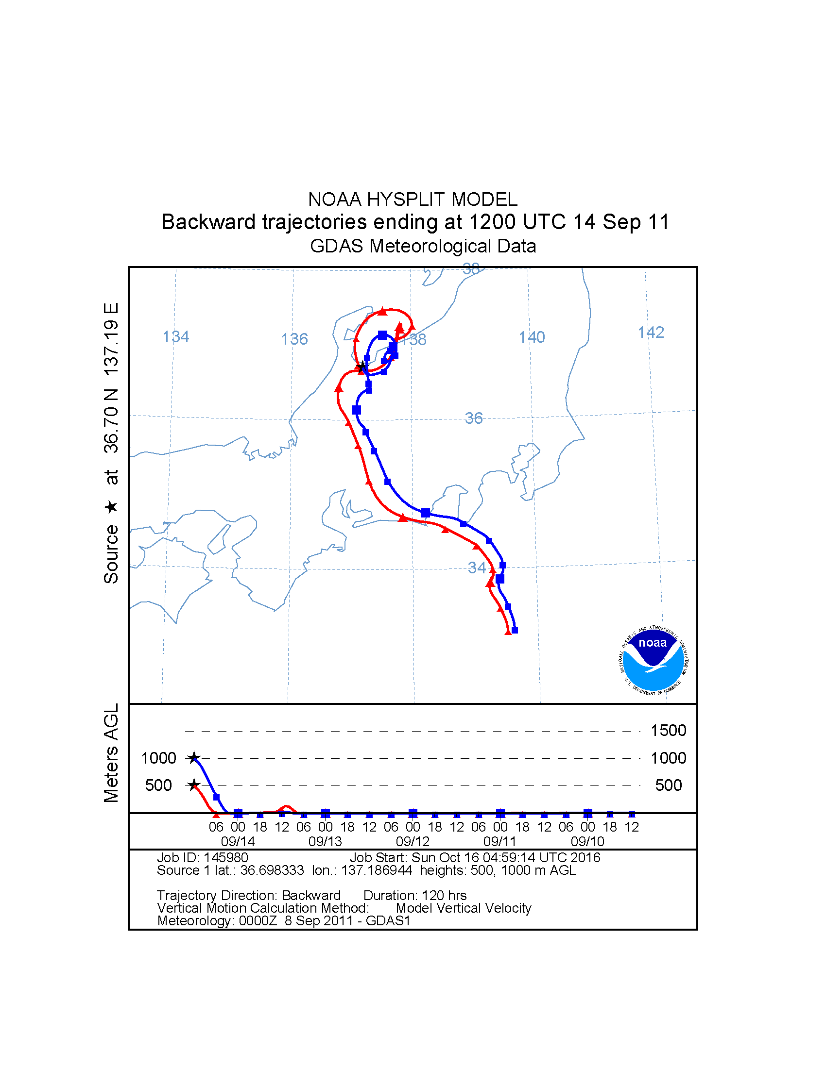


(J)

(I)

**Supplementary Figure 1. (continued)**


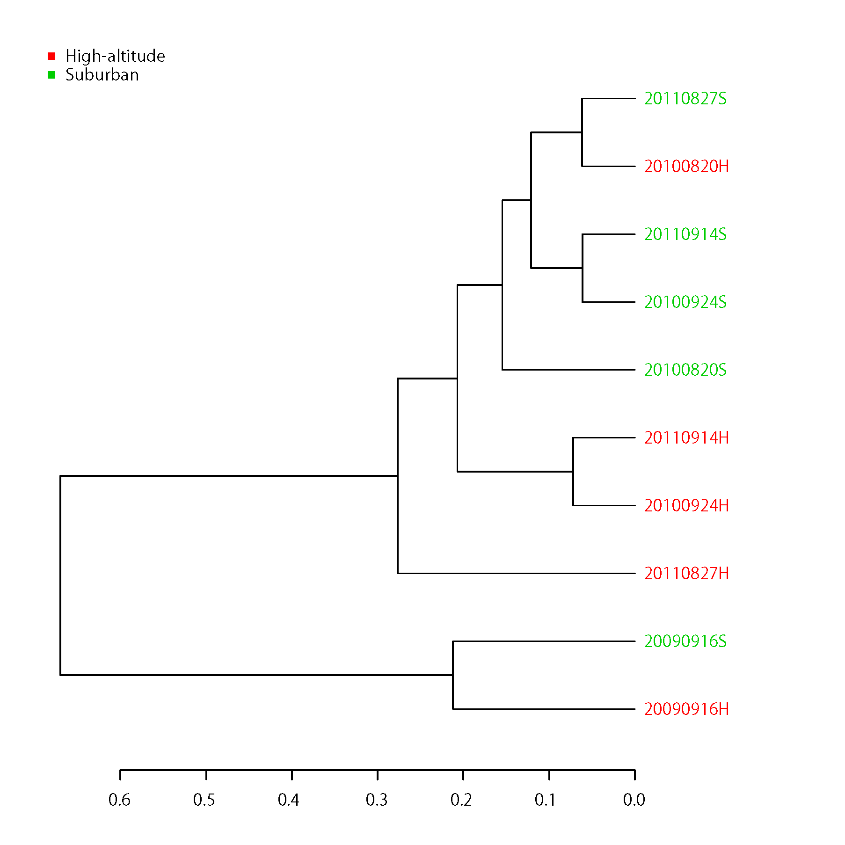
(A) bacterial community


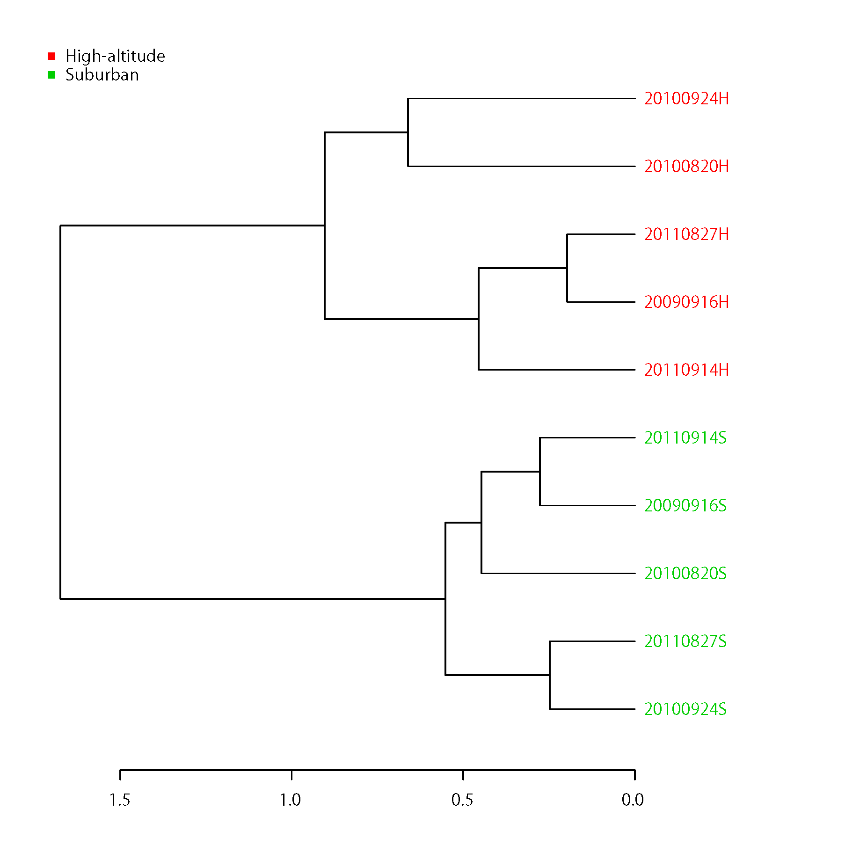
(B) eukaryotic community

**Supplementary Figure 2.** Hierarchical clustering of bacterial community (A) and eukaryotic community (B) based on the Bray-Curtis dissimilarity index.

1. **bacterial community**


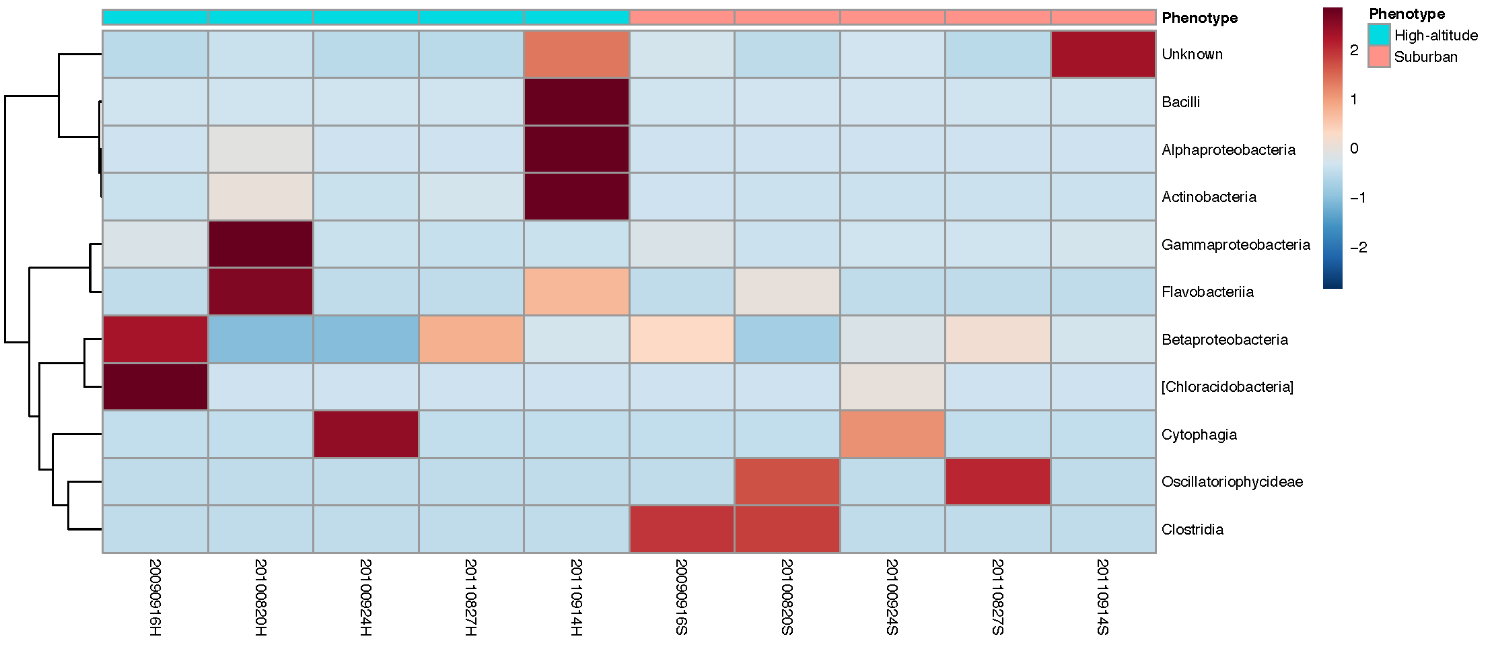


1. **eukaryotic community**


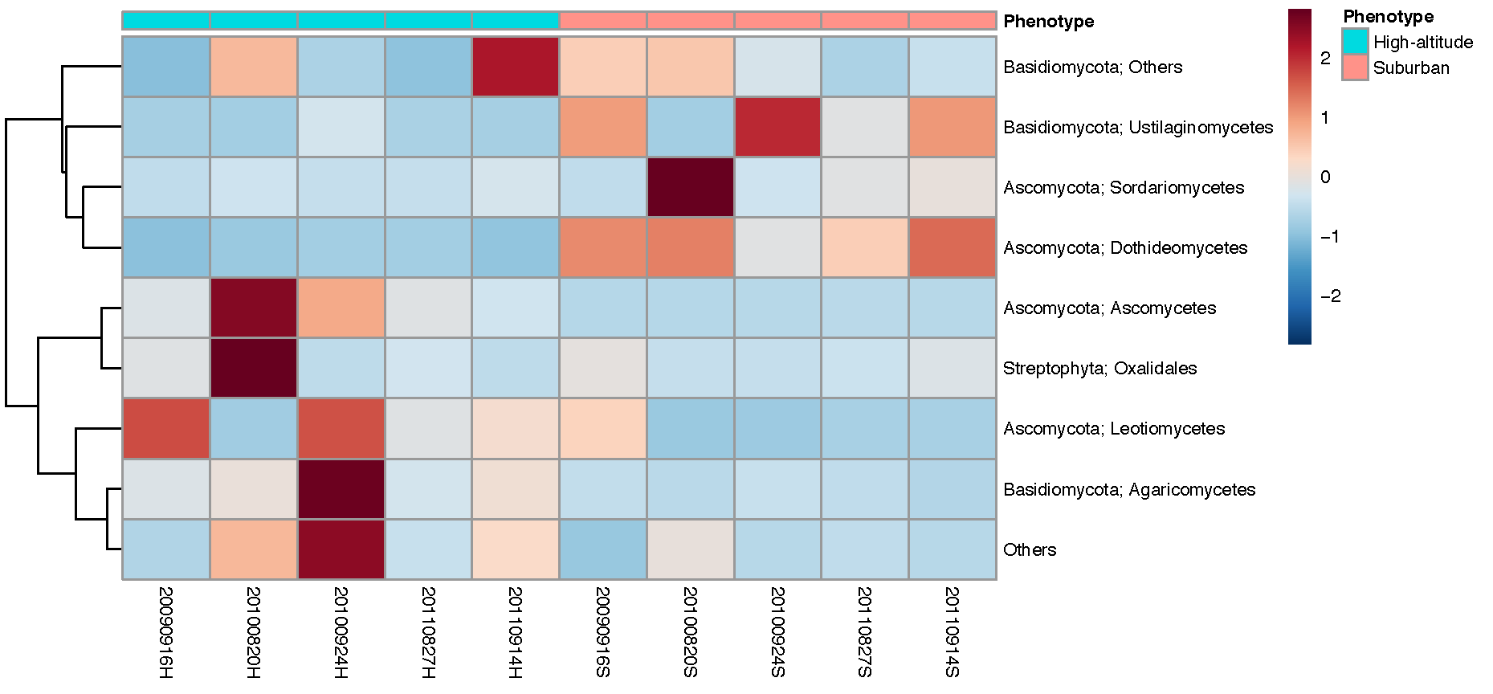


**Supplementary Figure 3.** Heatmap and dendrogram of abundant bacterial community (A) and eukaryotic community (B) classes present in samples.
